# Combining HJ CCD, GF-1 WFV and MODIS Data to Generate Daily High Spatial Resolution Synthetic Data for Environmental Process Monitoring

**DOI:** 10.3390/ijerph120809920

**Published:** 2015-08-20

**Authors:** Mingquan Wu, Wenjiang Huang, Zheng Niu, Changyao Wang

**Affiliations:** 1 The State Key Laboratory of Remote Sensing Science, Institute of Remote Sensing and Digital Earth, Chinese Academy of Sciences, Beijing 100101, China; E-Mails: niuzheng@radi.ac.cn (Z.N.); wangcy508@radi.ac.cn (C.W.); 2 Laboratory of Digital Earth Sciences, Institute of Remote Sensing and Digital Earth, Chinese Academy of Sciences, Beijing 100094, China; E-Mail: huangwj@radi.ac.cn

**Keywords:** HJ CCD, GF-1 WFV, STDFA, ESTARFM

## Abstract

The limitations of satellite data acquisition mean that there is a lack of satellite data with high spatial and temporal resolutions for environmental process monitoring. In this study, we address this problem by applying the Enhanced Spatial and Temporal Adaptive Reflectance Fusion Model (ESTARFM) and the Spatial and Temporal Data Fusion Approach (STDFA) to combine Huanjing satellite charge coupled device (HJ CCD), Gaofen satellite no. 1 wide field of view camera (GF-1 WFV) and Moderate Resolution Imaging Spectroradiometer (MODIS) data to generate daily high spatial resolution synthetic data for land surface process monitoring. Actual HJ CCD and GF-1 WFV data were used to evaluate the precision of the synthetic images using the correlation analysis method. Our method was tested and validated for two study areas in Xinjiang Province, China. The results show that both the ESTARFM and STDFA can be applied to combine HJ CCD and MODIS reflectance data, and GF-1 WFV and MODIS reflectance data, to generate synthetic HJ CCD data and synthetic GF-1 WFV data that closely match actual data with correlation coefficients (*r*) greater than 0.8989 and 0.8643, respectively. Synthetic red- and near infrared (NIR)-band data generated by ESTARFM are more suitable for the calculation of Normalized Different Vegetation Index (NDVI) than the data generated by STDFA.

## 1. Introduction

Coarse-resolution satellite data obtained, for example from the Advanced Very High Resolution Radiometer (AVHRR) [1], Systeme Pour l’Observation de la Terre (SPOT) Vegetation (VGT) [2], and from the Moderate Resolution Imaging Spectroradiometer (MODIS) [3], are widely used in areas such as land cover and land use mapping [4,5], crop mapping and yield forecasting [6,7], global change [8], vegetation trends and phenology estimations [9,10], disaster monitoring [11,12,13,14] and atmospheric environment [15,16,17] and water environment monitoring [18]. The return cycle of these satellites is one to two days, making them suitable for dynamic monitoring of land surface processes, particularly AVHRR data, which provides the longest time series among global satellite measurements [19]. However, the spatial resolutions of these data are lower than 250 m. When the size of land objects is smaller than the spatial resolution of images acquired by these sensors, the recorded signals are often a mixture of different land cover types. This makes these data difficult to apply to high spatial resolution surface process monitoring. Medium spatial resolution satellite data, such as data from Landsat and the Advanced Spaceborne Thermal Emission and Reflection Radiometer (ASTER), can also be used for dynamic monitoring of land surface processes. However, because of the long return cycles of these satellites (>16 days) and the influence of clouds, the rate at which these satellites can obtain useful data is very low [20], and it is difficult to use their data to monitor rapid changes in land surface processes. Therefore, these satellite data are often used only for annual dynamic analyses, including the spatiotemporal dynamic analysis of ecosystems such as wetlands [21,22], forests [23,24,25], water [26], crops [27] and cities [28], for monitoring plant phenology [29] and for land management [30,31]. There is a lack of satellite data with high enough spatial and temporal resolutions to monitor rapid changes in land surface processes.

A solution to this problem is to combine coarse and medium spatial resolution satellite data to generate synthetic satellite data with high spatial and temporal resolutions. This method is called spatial and temporal data fusion technology and several such approaches have recently been proposed. Gao *et al.* [32] introduced the Spatial and Temporal Adaptive Reflectance Fusion Model (STARFM) for blending MODIS and Landsat imagery. Several studies have applied STARFM, mainly in coniferous areas for urban environmental variable extraction, vegetated dry-land ecosystem monitoring, public health studies and daily land surface temperature generation [33,34,35,36,37]. Zhu *et al.* [38] enhanced STARFM for complex heterogeneous regions. Emelyanova *et al.* [39] assessed the accuracy of STARFM and ESTARFM (Enhanced STARFM) for two landscapes with contrasting spatial and temporal dynamics. Jarihani *et al.* [40] evaluated the accuracy of STARFM and ESTARFM to downscale MODIS indices to match the spatial resolution of Landsat. Scholars have also proposed methods based on linear mixed models [41,42,43]. Wu *et al.* [44] proposed a Spatial and Temporal Data Fusion Approach (STDFA) to calculate the real surface reflectance of fine-resolution pixels from the mean reflectance of each land cover class, disaggregated using unmixing methods. They also applied this method to the estimation of high spatial and temporal resolution Leaf Area Index [45] and land surface temperature [46] data. Gevaert and García-Haro [47] compared STARFM and an unmixing-based algorithm. STARFM and ESTARFM are more suitable for complex heterogeneous regions, while unmixing methods such as STDFA are more suitable for cases that downscale the spectral characteristics of medium-resolution input imagery [47].

The proposed spatial and temporal data fusion approaches mainly focus on the fusion of Landsat and MODIS data. However, with the recent launch of new satellites, there is a need to validate these methods for the new sensors. In recent years, China has launched two moderate-resolution satellites, Huanjing satellite (HJ), and Gaofen satellite no. 1 (GF-1). Wei *et al.* [48] compared the data quality of the HJ charge coupled device (CCD) and the Landsat Thematic Mapper (TM) sensor data. They found that the radiation accuracy, clarity and signal-to-noise ratio (SNR) of the HJ CCD data were lower than for the Landsat TM data. This issue is particularly important for Chinese satellite data because of the poor data quality.

To address this problem, the objectives of the present study are: (1) to validate the applicability of ESTARFM and STDFA to HJ and GF satellite data and (2) to analyse the influence of MODIS data of different spatial resolutions on the application of ESTARFM and STDFA.

**Figure 1:**
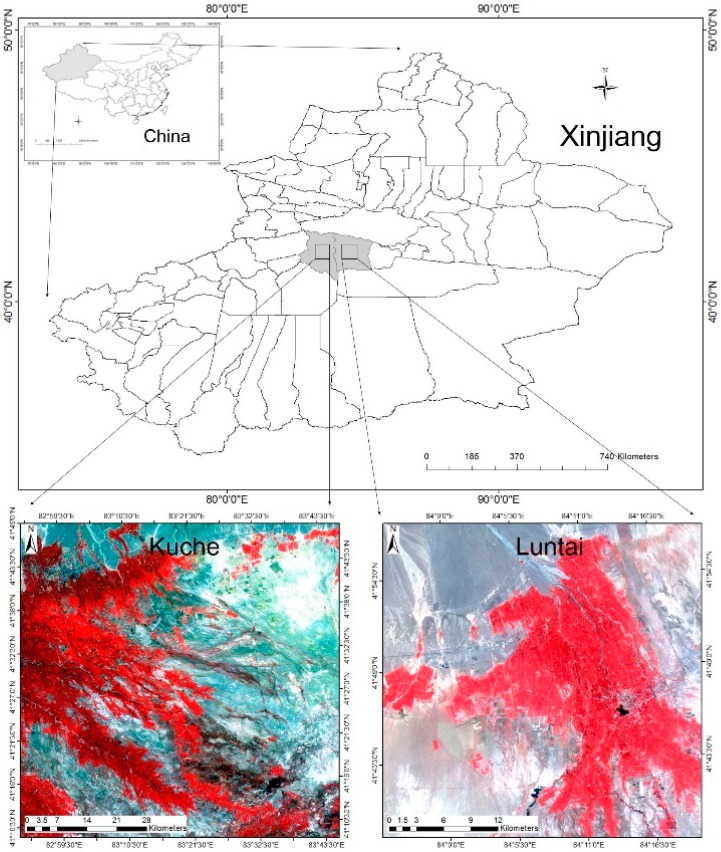
Locations of the study areas.

## 2. Study Area and Data Preparation

### 2.1. Study Area

Two counties located in Xinjiang Province, China, were selected as the study areas (Figure 1). The first county, Kuche, Aksu City, Xinjiang Province, China, is located at 40°46′–42°35′ N, 82°35′–84°17′ E. The second county, Luntai, Bayinguoleng Mongolian Autonomous Prefecture, Xinjiang Province, China, is located at 41°05′–42°32′ N, 83°38′–85°25′ E. Both counties are located in the south of the middle Tianshan Mountain and the north of Tarim Basin and belong to a warm temperate zone, with a continental arid climate. The north of both counties is mountainous, while the central and southern regions consist of plains. The main landforms of both counties are plains, which are mainly devoted to agriculture, cities and deserts.

### 2.2. Data and Pre-Processing

Three HJ CCD datasets, six GF-1 wide field of view camera (WFV) datasets and six MODIS surface reflectance datasets were used in this study (Table 1). All of these data were acquired during clear sky conditions and are of good quality.

**Table 1:** Images used in this research.

Study Area	Acquisition Date	Path/Row	
		HJ-1 CCD	GF-1 WFV
Kuche	03/10/2013	41/64	58/173
	07/10/2013	42/64	56/174
	15/10/2013	45/64	58/173
Luntai	03/10/2013	41/64	58/167
	07/10/2013	42/64	56/168
	15/10/2013	45/64	56/168

#### 2.2.1. HJ-1 CCD Data

The HJ satellite constellation consists of two satellites (HJ-1-A and HJ-1-B), and was launched on 6 September 2008. HJ-1-A is equipped with a CCD camera and a hyperspectral imager (HSI). HJ-1-B is equipped with a CCD camera and an infrared camera (IRS). The HJ satellite constellation can image the entire Earth at two-day intervals. Table 2 lists the sensors’ parameters for the HJ satellite constellation.

Three HJ CCD datasets were downloaded from the China Centre for Resources Satellite Data and Application (http://www.cresda.com/n16/index.html). These data were atmospherically corrected using the Fast Line-of-Sight Atmospheric Analysis of Spectral Hypercubes (FLAASH) Atmospheric Correction Model in the ENVI 5.0 software. The three HJ CCD datasets were then georeferenced using a second-order polynomial warping approach based on the selection of 45 ground control points (GCPs) from a 1:10,000 topographic map by the nearest neighbour resampling method, with a position error of within 0.67 HJ CCD pixels. 

**Table 2:** Parameters for HJ satellite constellation sensors.

Satellite	Sensor	Band	Bandwidth (nm)	Resolution (m)	Width (km)	Return Cycle (Days)
HJ-1-A	CCD	1	430–520	30	700	4
		2	520–600			
		3	630–690			
		4	760–900			
	HSI	128 bands	450–950	100	50	4
HJ-1-B	CCD	1	430–520	30	700	4
		2	520–600			
		3	630–690			
		4	760–900			
	IRS	5	750–1100	150	720	4
		6	1550–1750			
		7	3500–3900			
		8	10500–12500	300		

#### 2.2.2. GF-1 WFV Data 

The GF-1 satellite is a Chinese sun-synchronous, high-resolution satellite. It was launched on 26 April 2013. The GF-1 satellite is equipped with two types of sensors: two panchromatic multispectral sensors (MPS) and four WFV cameras. It can acquire panchromatic images with a resolution of 2 m, and multispectral images at a resolution of 8 m or 16 m. Table 3 lists the sensors’ parameters for the GF-1 satellite.

**Table 3:** Parameters for the GF-1 satellite sensors.

Sensor	Bandwidth (nm)	Resolution (m)	Width (km)	Return Cycle (Days)
MPS	450–900	2	60	4 (side swing) 41 (no side swing)
	430–520	8		
	520–600			
	630–690			
	760–900			
WFV	430–520	16	800	4
	520–600			
	630–690			
	760–900			

Six GF-1 WFV datasets, provided by the GF Satellite Application Technology Centre, Institute of Remote Sensing and Digital Earth, Chinese Academy of Sciences, were used in this study (Table 1). These data were orthorectified with ENVI 5.0 software, using rational polynomial coefficients (RPC) with a Digital Elevation Model (DEM) and 36 GCPs selected from a 1:10,000 topographic map. The data were then atmospherically corrected using the FLAASH Atmospheric Correction Model with the ENVI 5.0 software provided by Esri China Information Technology Co., Ltd., Beijing, China.

#### 2.2.3. MODIS Data

Three 500 m resolution daily MODIS surface reflectance products (MOD09GA) and three 250 m resolution daily MODIS surface reflectance products (MOD09GQ) were used in this study (Table 1). These MODIS images were reprojected from the native sinusoidal projection onto the UTM-WGS84 reference system, and were resized to the selected study area using MODIS Reprojection Tool (MRT) software. These MODIS data were then georeferenced using a second-order polynomial warping approach based on the selection of 28 or 32 GCPs from 500 m or 250 m GF-1 WFV images, respectively, with a nearest neighbour resampling method that has a position error of within 0.53 and 0.67 GF-1 WFV pixels, respectively. The 500 m and 250 m GF-1 WFV images were resized from georeferenced GF-1 WFV images using the pixel aggregate resampling method.

## 3. Methods

### 3.1. Model Introduction

#### 3.1.1. ESTARFM

ESTARFM was proposed to improve the STARFM algorithm for the accurate prediction of surface reflectance in heterogeneous landscapes using the observed reflectance trend between two points in time and spectral unmixing theory [38]. According to the linear mixture model, the changes in coarse-resolution reflectance from *t_0_* to *t_k_* can be expressed as:

(1) $$R\left(t_{k}\right)-R\left(t_{0}\right)=\sum_{i=1}^{M}\frac{f_{i}}{a}\left(r\left(t_{k}\right)-r\left(t_{0}\right)\right)$$

where $R\left(t_{k}\right)$ and $R\left(t_{0}\right)$ are the coarse-resolution reflectances from time *t_0_* to *t_k_*, $r\left(t_{k}\right)$ and $r\left(t_{0}\right)$ are the fine-resolution reflectances from time *t_0_* to *t_k_*, *M* is the total number of endmembers, $f_{i}$ is the fraction of the *i-*th land type and *a* is the sensor calibration coefficient. ESTARFM assumes that thechange in the reflectance of each land type is linear with time:

(2) $$r\left(t_{k}\right)=h_{k}\times \Delta t+r\left(t_{0}\right)$$

where $\Delta t=t_{k}-t_{0}$ and *h_k_* is the rate of change of the *k*-th endmember, which was assumed to be constant from time *t_0_* to *t_k_*. Then, Equations (1) and (2) can be rewritten as:

(3) Δt=(r(tk)−r(t0))hk

(4) $$R\left(t_{k}\right)-R\left(t_{0}\right)=\Delta t\sum_{i=1}^{M}\frac{f_{i}h_{i}}{a}$$

Substituting Equation (3) into Equation (4), the ratio, *v_k_*, of the change in reflectance for the *k*-th endmember to the change in reflectance for a coarse pixel can be described as:

(5) $$\frac{r\left(t_{k}\right)-r\left(t_{0}\right)}{R\left(t_{k}\right)-R\left(t_{0}\right)}=\frac{h_{k}}{\sum_{i=1}^{M}\frac{f_{i}h_{i}}{a}}=v_{k}$$

Equation (5) can be rewritten as:

(6) $$r\left(x,y,t_{k}\right)=r\left(x,y,t_{0}\right)+v_{k}\times \left(R\left(x,y,t_{k}\right)-R\left(x,y,t_{0}\right)\right)$$

where (*x,y*) is the position of the target pixels.

By introducing additional information from the neighbouringpixels to reduce the influence of land cover changes, surface heterogeneity and solar geometrybi-directional reflectance distribution function (BRDF) changes, a weighted ESTARFM can be determined as Equation (7):

(7) $$r(x,y,t_{k})=r(x_{i},y_{j},t_{0})+\sum_{i=1}^{w}\sum_{j=1}^{w}\sum_{k=1}^{n}W_{ijk}\times v_{ijk}\left(R\left(x_{i},y_{j},t_{k}\right)-R\left(x_{i},y_{j},t_{0}\right)\right)$$

where *w* is the size of the search window; $W_{ijk}$ is the weight determined by the spectral difference $S_{ijk}$ and the temporal difference $T_{ijk}$ between the fine- and low-resolution data, and by the location distance $D_{ijk}$ between the target pixel and the candidate pixel; *k* is the number of pixels (*x_i,_y_j_*) in window *w*. These parameters are calculated as follows:

(8) $$W_{i}=\left(1/D_{i}\right)/\sum_{i=1}^{n}\left(1/D_{i}\right)$$

(9) $$D_{i}=\left(1+S_{i}\right)\times d_{i}$$

(10) $$d_{i}=1+\sqrt{{\left(x-x_{i}\right)}^{2}+{\left(y-y_{i}\right)}^{2}}/\left(w/2\right)$$

(11) $$S_{i}=\frac{E\left[\left(r_{i}-E\left(r_{i}\right)\right)\times \left(R_{i}-E\left(R_{i}\right)\right)\right]}{\sqrt{D\left(r_{i}\right)}\times \sqrt{D\left(R_{i}\right)}}$$

#### 3.1.2. STDFA

STDFA is based on a linear mixing model that assumes the reflectance of each coarse spatial resolution pixel is a linear combination of the responses of each land cover class contributing to the mixture [49]:

(12) $$R(x,y,t)=\sum_{i=0}^{M}f_{i}(x,y,i)\times \bar{r}(i,t)+\xi (x,y,t)$$

$$\mathrm{Constraint}:\sum_{i=0}^{M}f_{i}(x,y,i)<1\;\mathrm{and}\;f_{i}(x,y,i)\geq 0\;\;\mathrm{for all}.$$

where $\bar{r}(i,t)$ is the mean reflectance of land type *i* at time *t* and ξ(x,y,t) is the residual error term. By inputting the fraction data $f_{i}(x,y,i)$ extracted from the land cover map, the mean reflectance for land cover class *i* can be calculated by solving Equation (12) using the ordinary least squares technique. Then, based on the assumption that the temporal variation properties of each fine-resolution pixel in the same class are constant, the STDFA predicts synthetic high-resolution spatial imagery as follows:

(13) $$r(x,y,t_{k})=r(x,y,t_{0})+\bar{r}(c,t_{k})-\bar{r}(c,t_{0})$$

### 3.2. Model Application

According to Equation (7), three images are needed for ESTARFM: fine-resolution data acquired at time *t_0_*, called the base image, and two low-resolution datasets acquired at times *t_0_* and *t_k_*, called the time series low-resolution data. Two pairs of fine-resolution and low-resolution datasets acquired at time *t_0_* and time *t_l_* are also required to calculate the spectral similarity index. According to Equation (12), three images are needed for STDFA: a fine-resolution dataset acquired at time *t_0_*, called the base image, and two low-resolution datasets acquired at times *t_0_* and *t_k_*, called the time series low-resolution data. Two fine-resolution datasets acquired at time *t_0_* and time *t_l_* are also required for classification. The outputs of ESTARFM and STDFA are synthetic fine-resolution data acquired at time *t_k_*. In this study, HJ-1 CCD and GF-1 WFV data acquired on 3 October 2013 were used as the base image. MODIS images acquired on 3 October 2013 and 7 October 2013 were used as the time series low-resolution data. Two pairs, HJ-1 CCD and MODIS data, or GF-1 WFV and MODIS data, acquired on 3 October 2013 and 15 October 2013 were used to calculate the spectral similarity index for ESTARFM. Two HJ-1 CCD or GF-1 WFV datasets, acquired on 3 October 2013 and 15 October 2013, were used for classification in STDFA. The outputs of ESTARFM and STDFA are synthetic HJ-1 CCD and GF-1 WFV data acquired on 7 October 2013.

### 3.3. Validation of Results

Since the objective of the ESTARFM and STDFA methods was to generate synthetic fine-resolution data, actual HJ-1 CCD and GF-1 WFV data acquired on 7 October 2013 were used to validate the algorithm using the methods proposed by Wu *et al.* [44]. First, the results were qualitatively evaluated using visual interpretation. The greater the similarity between the synthetic and actual fine-resolution data, the higher the accuracy of the model. Second, the results were quantitatively evaluated using the correlation analysis method. Parameters, such as the correlation coefficient (*r*), variance (Var), mean absolute difference (MAD), bias and root mean square error (RMSE), were calculated to quantitatively evaluate the precision of these models. A higher value of *r* and lower variance, MAD, bias and RMSE indicate higher accuracy.

### 3.4. Accuracy Comparison for the Fusion Results Using 250 m and 500 m MODIS Data

There are two reflectance MODIS data products: MOD09GQ and MOD09GA. Both products can be used in spatial and temporal data fusion, but the question remains as to whether the spatial resolution differences between these two products have an impact on the fusion accuracy. To answer this question, we applied STDFA and ESTARFM using MOD09GQ data in Kuche and Luntai, and compared the fusion results with results obtained using MOD09GA data. The correlation analysis method was used to evaluate the similarity of the actual fine-resolution data and the synthetic fine-resolution data generated by inputting MOD09GQ and MOD09GA. By comparing the *r*, variance, MAD, bias and RMSE we can analyse the influence of spatial resolution differences for these two data products.

### 3.5. Comparison of Actual NDVI and NDVI Calculated Using Synthetic Data

The synthetic red- and near infrared (NIR)-band data were generated using STDFA and ESTARFM in Kuche and Luntai, allowing Normalized Different Vegetation Index (NDVI) images in these two study areas to be calculated. The correlation analysis method was then used to evaluate the similarity of the NDVI image calculated from synthetic fine-resolution data and the NDVI image calculated from actual fine-resolution data. 

## 4. Results

### 4.1. HJ and MODIS Fusion Results

**Figure 2:**
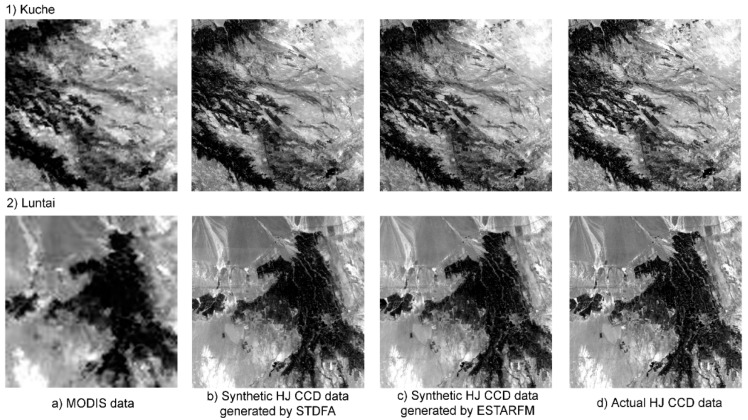
Comparison of MODIS, HJ CCD and synthetic data generated by the ESTARFM and STDFA acquired on 7 October 2013: (**a**) MODIS data; (**b**) synthetic HJ CCD data generated by STDFA; (**c**) synthetic HJ CCD data generated by ESTARFM; (**d**) actual HJ CCD data.

By inputting a base HJ CCD image, two days of MOD09GA data and two days of multi-spectral HJ CCD images, a synthetic multi-spectral HJ CCD image was generated by STDFA. By inputting a base HJ CCD image, two days of multi-spectral MOD09GA data and two days of multi-spectral MODIS data and HJ CCD datasets, a synthetic multi-spectral HJ CCD image was also generated by ESTARFM. These synthetic multi-spectral HJ CCD images contained four bands, including blue, green, red and NIR. Figure 2a shows the actual observed MODIS surface reflectance in the red band acquired on 7 October 2013 in Kuche and Luntai. Figure 2b, c show the synthetic surface reflectance imagery in the red band generated by STDFA and ESTARFM, respectively, for the two study areas. Figure 2d shows the actual observed HJ CCD red-band surface reflectance acquired on 7 October 2013 in Kuche and Luntai. 

The actual high spatial resolution data acquired on 7 October 2013 was used to evaluate the accuracy of these models. Through visual interpretation, we found that the synthetic and actual HJ CCD data are very similar, and were hard to distinguish between with the naked eye. Table 4 shows the results of the quantitative evaluation using the correlation analysis method. From Table 4, we can see that both STDFA and ESTARFM can generate synthetic HJ CCD data that are very similar to actual HJ CCD data, with r values greater than 0.8989. STDFA showed a slightly better performance than ESTARFM for Luntai and Kuche.

**Table 4:** Results of the correlation analysis between synthetic and actual HJ CCD data.

Kuche								
**Parameters**	**STDFA**				**ESTARFM**			
	Blue	Green	Red	NIR	Blue	Green	Red	NIR
*r*	0.9493	0.9642	0.9759	0.9231	0.9463	0.9601	0.9714	0.8989
Var	0.0001	0.0002	0.0002	0.0001	0.0002	0.0002	0.0002	0.0002
MAD	0.0091	0.0104	0.0096	0.0086	0.0093	0.0110	0.0106	0.0100
RMSE	0.0494	0.0422	0.0166	0.0364	0.0425	0.0305	0.0218	0.0177
bias	−0.0479	−0.0399	−0.0109	−0.0344	−0.0406	−0.0270	−0.0169	−0.0115
**Luntai**								
**Parameters**	**STDFA**				**ESTARFM**			
	Blue	Green	Red	NIR	Blue	Green	Red	NIR
*r*	0.9591	0.9640	0.9761	0.9608	0.9585	0.9598	0.9724	0.9475
Var	0.0002	0.0002	0.0002	0.0001	0.0002	0.0003	0.0002	0.0002
MAD	0.0098	0.0114	0.0102	0.0081	0.0099	0.0119	0.0108	0.0108
RMSE	0.0461	0.0404	0.0336	0.0226	0.0466	0.0344	0.0275	0.0175
bias	−0.0441	−0.0375	−0.0308	−0.0197	−0.0446	−0.0305	−0.0235	−0.0098

### 4.2. GF and MODIS Fusion Results

**Figure 3:**
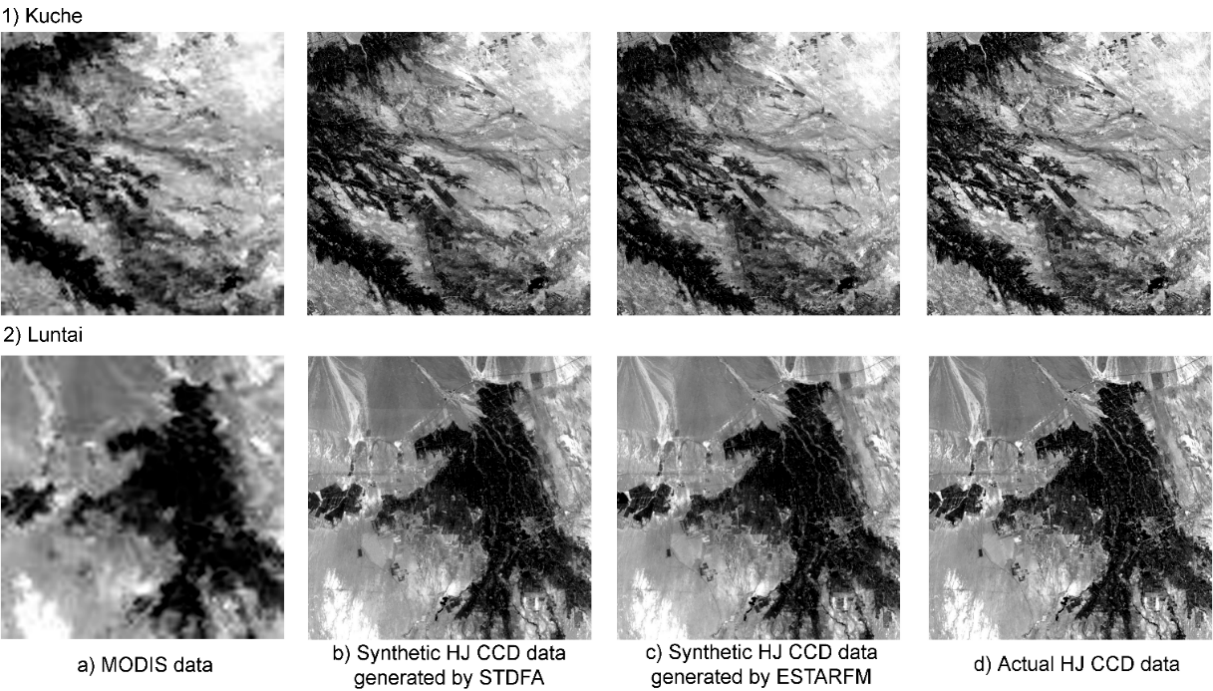
Comparison of MODIS, GF-1 WFV and synthetic data generated by ESTARFM and STDFA acquired on 7 October 2013: (**a**) MODIS data; (**b**) synthetic GF-1 WFV data generated by STDFA; (**c**) synthetic GF-1 WFV data generated by ESTARFM; (**d**) actual GF-1 WFV data.

By inputting a base GF-1 WFV image, two days of MOD09GA data and two days of multi-spectral GF-1 WFV images, a synthetic multi-spectral GF-1 WFV image was generated by STDFA. By inputting a base GF-1 WFV image, two days of multi-spectral MOD09GA data and two days of multi-spectral MODIS data and GF-1 WFV datasets, a synthetic multi-spectral GF-1 WFV image was also generated by ESTARFM. These synthetic multi-spectral GF-1 WFV images contained four bands, including blue, green, red and NIR. Figure 3a shows the actual observed MODIS surface reflectance in the red band acquired on 7 October 2013 in Kuche and Luntai. Figure 3b, c show the synthetic GF-1 WFV surface reflectance imagery in the red band generated by STDFA and ESTARFM, respectively, for the two study areas. Figure 3d shows the actual observed GF-1 WFV red-band surface reflectance acquired on 7 October 2013 in Kuche and Luntai.

Through visual interpretation, we found that the synthetic and actual GF-1 WFV data are very similar and were not distinguishable with the naked eye. Table 5 shows the results of the quantitative evaluation using the correlation analysis method. From Table 5, we also find that both STDFA and ESTARFM can generate synthetic GF-1 WFV data that are very similar to actual GF-1 WFV data, with r values greater than 0.8643. ESTARFM performed slightly better than STDFA in Kuche, while the accuracies of these two methods were very similar in Luntai.

**Table 5:** Results of the correlation analysis between synthetic and actual GF-1 WFV data.

Kuche								
**Parameters**	**STDFA**				**ESTARFM**			
	Blue	Green	Red	NIR	Blue	Green	Red	NIR
*r*	0.9658	0.9687	0.9778	0.9513	0.9669	0.9705	0.9790	0.9563
Var	0.0001	0.0001	0.0002	0.0001	0.0001	0.0001	0.0001	0.0001
MAD	0.0079	0.0089	0.0093	0.0081	0.0076	0.0085	0.0088	0.0078
RMSE	0.0264	0.0279	0.0254	0.0222	0.0220	0.0209	0.0180	0.0168
bias	−0.0240	−0.0252	−0.0222	−0.0188	−0.0193	−0.0174	−0.0135	−0.0124
**Luntai**								
**Parameters**	**STDFA**				**ESTARFM**			
	Blue	Green	Red	NIR	Blue	Green	Red	NIR
*r*	0.9334	0.9199	0.9315	0.8654	0.9338	0.9251	0.9314	0.8643
Var	0.0002	0.0003	0.0004	0.0006	0.0002	0.0003	0.0004	0.0006
MAD	0.0083	0.0105	0.0102	0.0098	0.0082	0.0096	0.0102	0.0105
RMSE	0.0287	0.0245	0.0287	0.0317	0.0272	0.0274	0.0261	0.0289
bias	−0.0249	−0.0165	−0.0211	−0.0195	−0.0231	−0.0211	−0.0175	−0.0141

### 4.3. Accuracy Comparison for the Fusion Results Using 250 m and 500 m MODIS Data

To analyse the influence of spatial resolution differences, we applied STDFA and ESTARFM using MOD09GQ data in Kuche and Luntai, and compared the fusion results with the results obtained using MOD09GA data. Table 6 shows the results of the correlation analysis between synthetic data generated using MOD09GQ data and actual data. Comparing Table 6, Table 4 and Table 5, we find that the spatial resolution differences had more impact on the fusion accuracy for ESTARFM than for STDFA in these two study areas. This is mainly because the MODIS reflectance was directly used to calculate the fine reflectance in ESTARFM, while it was used to calculate the mean reflectance of each land cover type in STDFA.

**Table 6:** Results of the correlation analysis between synthetic data generated using MOD09GA data and actual data.

GF-1 WFV								
Study Area	Kuche				Luntai			
**Parameters**	**STDFA**		**ESTARFM**		**STDFA**		**ESTARFM**	
	Red	NIR	Red	NIR	Red	NIR	Red	NIR
r	0.9785	0.9489	0.9776	0.9505	0.9306	0.8673	0.9302	0.8624
Var	0.0001	0.0001	0.0001	0.0001	0.0004	0.0006	0.0004	0.0006
MAD	0.0091	0.0081	0.0089	0.0084	0.0099	0.0096	0.0108	0.0108
RMSE	0.0253	0.0269	0.0192	0.0186	0.0286	0.0314	0.0248	0.0279
bias	−0.0223	−0.0243	−0.0151	−0.0142	−0.0212	−0.0193	−0.0152	−0.0116
**HJ CCD**								
**Study Area**	**Kuche**				**Luntai**			
**Parameters**	**STDFA**		**ESTARFM**		**STDFA**		**ESTARFM**	
	Red	NIR	Red	NIR	Red	NIR	Red	NIR
r	0.9783	0.9186	0.9690	0.8818	0.9737	0.9619	0.9533	0.9728
Var	0.0001	0.0001	0.0002	0.0002	0.0002	0.0001	0.0002	0.0002
MAD	0.0093	0.0089	0.0111	0.0108	0.0097	0.0080	0.0102	0.0108
RMSE	0.0360	0.0363	0.0219	0.0174	0.0335	0.0223	0.0173	0.0277
bias	−0.0338	−0.0343	−0.0167	−0.0099	−0.0309	−0.0194	−0.0109	−0.0237

### 4.4. Comparison of Actual NDVI and NDVI Calculated Using Synthetic Data

NDVI images calculated using actual data were used to evaluate the quality of the synthetic NDVI images. Table 7 shows the results of the correlation analysis between synthetic and actual NDVI data. From Table 7, we find that the NDVI data calculated using synthetic data generated by ESTARFM were more similar to actual NDVI than the NDVI data calculated using synthetic data generated by STDFA.

**Table 7:** Result of the correlation analysis between synthetic and actual NDVI data.

**Parameters**	**GF-1 WFV**				**HJ CCD**			
	**Kuche**		**Luntai**		**Kuche**		**Luntai**	
	STDFA	ESTARFM	STDFA	ESTARFM	STDFA	ESTARFM	STDFA	ESTARFM
*r*	0.9798	0.9803	0.9703	0.9742	0.9686	0.9722	0.9769	0.9801
Var	0.0012	0.0011	0.0017	0.0016	0.0011	0.0011	0.0017	0.0016
MAD	0.0264	0.0236	0.0266	0.0259	0.0252	0.0255	0.0318	0.0315
RMSE	0.0463	0.0372	0.0479	0.0461	0.0416	0.0405	0.0580	0.0568
bias	0.0300	0.0180	0.0247	0.0226	−0.0247	0.0223	0.0412	0.0400

## 5. Discussion

This study applied and demonstrated the use of ESTARFM and STDFA in combining HJ CCD, GF-1 WFV and MODIS data to generate high spatial resolution data. Although the data quality of HJ CCD data is lower than Landsat data, both the ESTARFM and STDFA algorithms can generate daily synthetic high spatial resolution data accurately, with r values higher than 0.8643 and RMSEs lower than 0.0360. As the MODIS sensor can acquire daily images, ESTARFM and STDFA can be used to enhance the proportion of useful HJ CCD and GF-1 WFV data. For example, the proportion of useful MODIS data in Luntai for 2013 is 48.49%, while the proportions of useful HJ CCD data and useful GF-1 WFV data are 15.07% and 10.29%, respectively. Owing to the high proportion of useful data, this method is potentially useful for high spatial resolution environmental process monitoring, and can be applied in natural resource damage assessments and environmental policy and management. However, there are some issues should be addressed in the application of this method:

(1) Influence of the satellite data quality. The test areas for the HJ-MODIS fusion and the GF-MODIS fusion in Kuche were the same, while the test area for the GF-MODIS fusion was a little larger than the area for the HJ-MODIS fusion in Luntai. Therefore, we can only analyse the influence of the satellite data quality on the data fusion for the Kuche area. As an early Chinese moderate-resolution satellite, the quality of HJ satellite data is lower than the quality of GF-1 satellite data. The position error of HJ CCD data is greater than 1 km, while the position error of GF-1 WFV data is less than 100 m. Furthermore, the SNR of HJ CCD data is much lower than the SNR of GF-1 WFV data. Table 6 lists the differences between Table 5 and Table 4, showing the influence of the sensor differences on the model accuracy. From Table 8, we can see that the influence of the sensor differences for STDFA is less significant than for ESTARFM. This is because STDFA had better noise immunity. Similar results were found for the fusion of ASTER and MODIS land surface temperature products [46].

**Table 8:** Influence of sensor differences on model accuracy.

**Parameters**	**STDFA**				**ESTARFM**			
	Blue	Green	Red	NIR	Blue	Green	Red	NIR
*r*	0.0165	0.0045	0.0018	0.0283	0.0206	0.0104	0.0077	0.0574
Var	0.0000	0.0000	0.0000	0.0000	0.0000	−0.0001	0.0000	0.0000
MAD	−0.0012	−0.0015	−0.0003	−0.0005	−0.0017	−0.0025	−0.0018	−0.0022
RMSE	−0.0230	−0.0143	0.0088	−0.0142	−0.0205	−0.0096	−0.0038	−0.0009
bias	−0.0238	−0.0147	0.0113	−0.0156	−0.0213	−0.0096	−0.0034	0.0009

**Figure 4:**
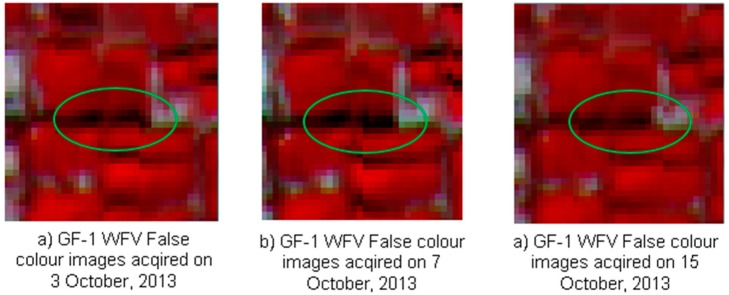
An example of the difference in shadow direction and length.

(2) Influence of bidirectional reflectance distribution function (BRDF) changes. For different data acquisition dates, the solar and satellite azimuth and zenith angles are different (Table 9). This leads to changes in the BRDF and hence in the information received by the sensor. More seriously, this could lead to differences in the shadow direction and length for the same object on different dates. Figure 4 shows an example of the difference in the shadow direction and length for trees on 3 October 2013, 7 October 2013 and 15 October 2013 recorded by the GF-1 WFV sensor. In the shaded area, the differences of reflectance in the NIR band between actual and synthetic GF-1 WFV data can reach 0.0676 for STDFA and 0.0638 for ESTARFM. However, in the unshaded area, the differences of reflectance in the NIR band between actual and synthetic GF-1 WFV data were only 0.0078 for STDFA and 0.0153 for ESTARFM. Therefore, we conclude that further study is required regarding this problem.

**Table 9:** Sensors and solar azimuth information from GF-1 WFV data.

Image Information	3 October 2013	7 October 2013	15 October 2013
Receive Time (local time)	13:34:11	13:33:24	13:31:47
Solar Azimuth	166.147	166.601	167.415
Solar Zenith	42.4117	40.9225	37.9947
Satellite Azimuth	235.912	121.314	106.892
Satellite Zenith	89.1838	88.3384	84.7204

(3) The ESTARFM and STDFA algorithms were proposed to enhance the temporal resolution of high spatial resolution data. High spatial and temporal remote sensing data can be generated using these methods. However, spectral fusion was not considered for these methods. Therefore, these methods can only be used for spatial and temporal fusion, and we recommend using the spectral fusion method in the future to improve the spectral resolution of sensors [50]. In addition, it has been proposed that these methods could be used to fuse optical images that are easily affected by cloudy weather. Although these methods can improve the proportion of useful data, a significant proportion of the data will be polluted by cloud. The development of an optical and radar data fusion algorithm is therefore an important direction for multi-source remote sensing data studies.

## 6. Conclusions

In two study areas located in Xinjiang Province, China, the ESTARFM and STDFA methods were applied to combine HJ CCD, GF-1 WFV and MODIS reflectance data. We found the following conclusions:

(1) Both ESTARFM and STDFA methods can be applied to combine HJ CCD and MODIS reflectance data, and GF-1 WFV and MODIS reflectance data. Synthetic HJ CCD data are very similar to actual HJ CCD data, with *r* values greater than 0.8989 generated using these two methods. Synthetic GF-1 WFV data are very similar to actual GF-1 WFV data, generating *r* values greater than 0.8643.
(2) The accuracy differences between the fusion of HJ CCD and MODIS data and the fusion of GF-1 WFV and MODIS data were lower for STDFA than for ESTARFM. STDFA had better noise immunity than ESTARFM.
(3) The spatial resolution differences between MOD09GQ and MOD09GA had a more significant impact on the fusion accuracy for ESTARFM than for STDFA.
(4) Synthetic red- and NIR-band data generated by ESTARFM are more suitable for the calculation of NDVI than the data generated by STDFA.
